# A *Staphylococcus aureus clpX* Mutant Used as a Unique Screening Tool to Identify Cell Wall Synthesis Inhibitors that Reverse *β*-Lactam Resistance in MRSA

**DOI:** 10.3389/fmolb.2021.691569

**Published:** 2021-06-04

**Authors:** Kristoffer T. Bæk, Camilla Jensen, Maya A. Farha, Tobias K. Nielsen, Ervin Paknejadi, Viktor H. Mebus, Martin Vestergaard, Eric D. Brown, Dorte Frees

**Affiliations:** ^1^ Department of Veterinary and Animal Sciences, Faculty of Health and Medical Sciences, University of Copenhagen, Copenhagen, Denmark; ^2^ Department of Biochemistry and Biomedical Sciences, Michael G. DeGroote Institute for Infectious Disease Research, McMaster University, Hamilton, ON, Canada

**Keywords:** ClpX, *Staphylococcus aureus*, cell wall synthesis, teichoic acid inhibitors, high-throughput screen, pathway-directed drug discovery, *β*-lactam antibiotics

## Abstract

*Staphylococcus aureus* is a leading cause of bacterial infections world-wide. Staphylococcal infections are preferentially treated with *β*-lactam antibiotics, however, methicillin-resistant *S. aureus* (MRSA) strains have acquired resistance to this superior class of antibiotics. We have developed a growth-based, high-throughput screening approach that directly identifies cell wall synthesis inhibitors capable of reversing *β*-lactam resistance in MRSA. The screen is based on the finding that *S. aureus* mutants lacking the ClpX chaperone grow very poorly at 30°C unless specific steps in teichoic acid synthesis or penicillin binding protein (PBP) activity are inhibited. This property allowed us to exploit the *S. aureus clpX* mutant as a unique screening tool to rapidly identify biologically active compounds that target cell wall synthesis. We tested a library of ∼50,000 small chemical compounds and searched for compounds that inhibited growth of the wild type while stimulating growth of the *clpX* mutant. Fifty-eight compounds met these screening criteria, and preliminary tests of 10 compounds identified seven compounds that reverse *β*-lactam resistance of MRSA as expected for inhibitors of teichoic acid synthesis. The hit compounds are therefore promising candidates for further development as novel combination agents to restore *β*-lactam efficacy against MRSA.

## Introduction

There is an unmet need for novel antibiotics to tackle the challenges associated with the world-wide dissemination of antibiotic resistant bacterial pathogens such as methicillin resistant *Staphylococcus aureus* (MRSA) (Tacconelli et al., 2018; Vestergaard et al., 2019). A common approach for identification of compounds with antibacterial activity is to screen large libraries of small molecules for compounds that inhibit bacterial growth. Whole cell screens based on growth inhibition are easily carried out in a high-throughput format, however, a major disadvantage of whole cell screens is that target identification is often challenging and time-consuming (French et al., 2017). In addition, whole cell screens for growth inhibition typically generate large numbers of active compounds, many of which have non-specific activities (Silver, 2011). Therefore, including a counter-screen that facilitates exclusion of non-specific inhibitors and allows identification of compounds targeting specific pathways early in the screening workflow can speed up the screening process tremendously (French et al., 2017; Buss et al., 2018).

In this report, we describe the development of a counter-screen that enables identification of compounds targeting cell wall synthesis in the major human pathogenic bacterium, *S. aureus*. The screen is based on growth (measured as change in absorbance) of an *S. aureus* mutant that lacks the ClpX chaperone, and the screen is therefore well suited for a high-throughput approach.

In all living cells, molecular chaperones are essential for facilitating folding and unfolding of proteins (Olivares et al., 2016). ClpX is a highly conserved ATP-dependent unfoldase that can associate with ClpP proteolytic subunits to form the ClpXP protease (Baker and Sauer, 2012). In *S. aureus*, deletion of the *clpX* gene confers a cold-sensitive phenotype characterized by severely reduced final yield at 30°C (Frees et al., 2003; Bæk et al., 2016). Remarkably, the poor growth of *S. aureus clpX* mutants can be rescued by inhibiting specific steps in the biosynthesis pathway of peptidoglycan or teichoic acids, the two major components of the Gram-positive cell wall (Bæk et al., 2016; Jensen et al., 2019). For example, *β*-lactam antibiotics, which inhibit cross-linking of peptidoglycan by binding irreversibly to the penicillin-binding proteins (PBPs), increase the growth yield of the *S. aureus clpX* mutant up to six times when added at sub-lethal concentrations (Jensen et al., 2019). Similarly, the antibiotics tunicamycin and tarocin A1 which both inhibit the TarO enzyme in the wall teichoic acid (WTA) biosynthesis pathway rescue growth of the *clpX* mutant, whereas other classes of antibiotics with different cellular targets, or inhibiting other steps in WTA or peptidoglycan synthesis have no effect (Jensen et al., 2019). Moreover, growth of *S. aureus clpX* mutants can be rescued genetically by inactivating the lipoteichoic acid synthase (LtaS) that catalyzes the last step in lipoteichoic acid (LTA) biosynthesis, as revealed by the characterization of spontaneous suppressor mutations acquired by *S. aureus clpX* strains (Bæk et al., 2016). LTA biosynthesis, similarly to WTA synthesis, is conditionally essential and an attractive target for novel antibiotics (Richter et al., 2013; Sewell and Brown, 2014; Coe et al., 2019).

Based on these findings we reasoned that an *S. aureus clpX* mutant could work as a screening tool to identify antimicrobial compounds targeting cell wall synthesis of *S. aureus*. Compounds that rescue growth of the *S. aureus clpX* mutant are predicted to inhibit crosslinking of peptidoglycan, or to inhibit specific steps in LTA synthesis, or WTA synthesis. Importantly, a number of elegant studies demonstrated that MRSA strains are sensitized to *β*-lactams if WTA or LTA biosynthesis is inhibited (Campbell et al., 2011; Farha et al., 2013, Roemer et al., 2013, Lee et al., 2016). Therefore, screened out compounds with a target in teichoic acid biosynthesis would have potential to be used in combination with *β*-lactams for treatment of MRSA-infections.

To test this hypothesis we set up the screening platform as follows. First, we identified compounds that inhibit growth of *S. aureus* from a library of 50,000 small chemical compounds. Second, the subset of *S. aureus* active compounds was deployed in the counter-screen to identify compounds that improve the growth yield of the *S. aureus clpX* mutant. From the initial 50,000 compounds, we identified 828 compounds with antimicrobial activity against *S. aureus*, and 58 of these enhanced growth of the *clpX* mutant indicating that they target cell wall synthesis. Finally, a subset of ten compounds was further tested, and seven out of seven hit compounds sensitized an MRSA strain to *β*-lactam antibiotics, demonstrating the power of the screen at identifying compounds that can restore antibiotic sensitivity in MRSA.

## Methods

### Bacterial Strains and Growth Conditions

*S. aureus* strains used in this study were the methicillin sensitive clinical isolate, SA564 (Somerville et al., 2002), SA564 *clpX* (Jelsbak et al., 2010) and the MRSA strains USA300 JE2 (Fey et al., 2013), and COL (Dyke et al., 1966). *S. aureus* strains were cultured in tryptic soy broth [TSB (Oxoid)] at 37 or 30°C with eration, or on TSB medium solidified with 1.5% (wt/vol) agar (TSA). When inoculating the *clpX* deletion strain, care was taken to avoid visibly larger colonies containing potential suppressor mutants (Bæk et al., 2016).

### Primary Screen

Screening for *S. aureus* growth inhibition was performed in 384-well microtiter plates (catalog no. 3701, Corning) in duplicate using a stand-alone Biomek FXP integrated liquid handler (Beckman Coulter). The screening library consisted of 50,000 small drug-like chemical compounds from the Maybridge screening collection (ThermoFisher). The evening before screening, a single colony of wild type *S. aureus* SA564 was inoculated into 5 ml of TSB and grown overnight at 37°C. On the day of screening, the overnight culture was diluted 1:200 in TSB and grown to mid-exponential phase (OD_600_ of ∼0.5). Cells were then diluted into fresh TSB to a final OD_600_ of 0.001. The Biomek FXP liquid handler was used to dispense in duplicate 20 μl of TSB followed by 0.4 μl of each compound of the 50,000 small-molecule library (1 mM stock dissolved in 100% DMSO) into each well. The liquid handler was then subsequently used to dispense 20 μl of culture (*S. aureus* SA564 OD_600_ 0.001), giving a final screening concentration of 10 μM. 1% DMSO, and 1% DMSO + 2.5 mg L^−1^ erythromycin were used as high and low controls, respectively. Plates were incubated at 37°C in a Cytomat stationary incubator (ThermoFisher) for 8 h. These conditions resulted in a Z’ value of 0.8 (Supplementary Figure S1A). After incubation, absorbance was read at 600 nm using an EnVision plate reader (PerkinElmer). Data were normalized to take into account both plate and well positional effects using a method previously described (Mangat et al., 2014). A statistical cutoff of 3 standard deviations below the mean of the data set was established to select active compounds.

To confirm the activity of the 993 selected *S. aureus* active compounds, a half-log serial dilution series (50 nM–5 mM) of each compound was prepared in DMSO. 1 µl of each dilution was dispensed in duplicate into dry wells on 96-well microtiter plates (catalog no. 3370, Corning) using a Biomek FX liquid handler, and a Freedom EVO liquid handler (Tecan) was then used to dispense 99 µl of culture (*S. aureus* SA564 OD_600_ = 0.001) prepared as described above, giving a final concentration range of 0.5 nM–50 µM. Eight 1% DMSO wells were included on each plate as no-compound controls. Plates were then incubated at 37°C with shaking (600 rpm) for 7 h. After incubation, absorbance was read at 600 nm using an Infinite M1000 Pro plate reader (Tecan). To take into account plate positional effects, data for each plate were normalized to the mean of the DMSO wells excluding the two lowest and two highest values. The dose-response relationship of 828 of the compounds resulted in a typical sigmoidal semi-logarithmic curve associated with growth inhibition. 165 compounds failed to inhibit growth in this assay and were discarded from the downstream analyses.

### Counter Screen: Growth Stimulation of *S. aureus* *clpX* Mutant

The evening before screening, a single small colony of *S. aureus* SA564 *clpX* was picked from a plate incubated at 37°C and inoculated into 1 ml TSB that was then incubated overnight at 37°C. On the day of screening, the overnight culture was diluted 1:200 in TSB and grown to mid-exponential phase (OD_600_ of 0.3–0.6) at 37°C. Cells were diluted into fresh TSB to a final OD_600_ of 0.1, and then diluted 1:10,000 into 400 ml TSB. A half-log serial dilution series (50 nM–5 mM) of each *S. aureus* active compound was prepared in DMSO, and 1 µl of each dilution was dispensed in duplicate into dry wells on 96-well microtiter plates (catalog no. 3370, Corning) using a Biomek FX liquid handler. A Freedom EVO liquid handler (Tecan) was then used to dispense 99 µl of the prepared *S. aureus clpX* culture, giving a final concentration range of 0.5 nM–50 µM. Eight 1% DMSO wells were included on each plate as no-compound controls. Plates were then incubated at 30°C with shaking (600 rpm) for 24 h. After incubation, absorbance was read at 600 nm using an Infinite M1000 Pro plate reader (Tecan). To take into account plate positional effects, data for each plate were normalized to the mean of the DMSO wells excluding the two lowest and two highest values. For each compound and each dose, the lowest normalized OD value of replicates 1 and 2 was used to determine an increase in final growth yield, and the highest of these values for each compound across all doses was used as the screen read-out. A compound was classified as active if this value was 1.5 or higher.

### Disk Diffusion Assay

The MRSA strain COL was inoculated on TSA plates and incubated at 37°C overnight. The next day, bacterial colonies were suspended in 0.9% NaCl, adjusted to 0.5 McFarland (Sensititre® nephelometer and the Sensititre® McFarland Standard), and streaked on TSA plates with or without the following compounds: BTB 00921 (4 mg L^−1^), HTS 01632 (6 mg L^−1^), BTB 04965 (3 mg L^−1^), S 14042 (3 mg L^−1^), SEW 02456 (6 mg L^−1^), AW 00778 (6 mg L^−1^), SPB 06643 (5 mg L^−1^), HTS 09153 (2 mg L^−1^), SPB 06551 (0.2 mg L^−1^), and JP 00945 (2 mg L^−1^). The plates were allowed to dry prior to the addition of antibiotic susceptibility discs (Oxoid) and incubated at 37°C for 24 h. The tested antibiotics were ampicillin (AMP; 10 µg), cefaclor (CEC; 30 µg), cefotaxime (CTX; 30 µg), cefoxitin (FOX; 30 µg), cefuroxime (CXM; 30 µg), cephazolin (KZ; 30 µg), ceftriaxone (CRO; 5 µg), ceftazidime (CAZ; 30 µg), cloxacillin (OB; 5 µg) imipenem (IPM; 10 µg), oxacillin (OX; 1 µg), penicillin G (P; 10 µg), and vancomycin (VA; 30 µg). The ratio of the diameters of the inhibition zones in the presence and absence of compound was used to calculate a sensitizing score for each compound/*β*-lactam combination: no change in the diameter of the inhibition zones was scored as 0, a <3-fold increase in the diameter of the inhibition zone in the presence of compound was scores as 1, while a 3–6 fold increase in the inhibition zone was scored as 2, and an increase of >6-fold was assigned a score of 3. The sensitizing scores in Table 1 were obtained by adding the single scores for each compound across all *β*-lactams.

**TABLE 1:** Hit-compounds listed according to their ability to increase the growth yield of SA564 *clpX*.

Compound	Structure	MIC (mg L^−1^) SA564/JE2	Stimulation fold^a^ follow-up (original screen)	β-lactam sensitizing score^b^
BTB 00921	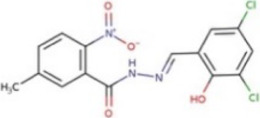	>32/>32	3.7 (1.7)	8
HTS 01632	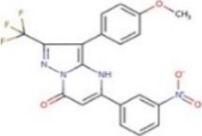	8–16/8–16	3.6 (3.6)	13
BTB 04965	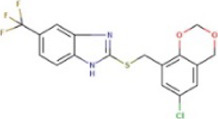	25/25	3.1 (2.7)	20
S 14042	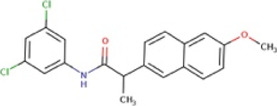	>25/>25	2.8 (3.2)	6
SEW 02456	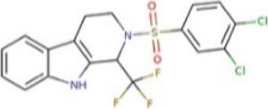	4/8^c^	2.3 (3.5)	6
AW 00778	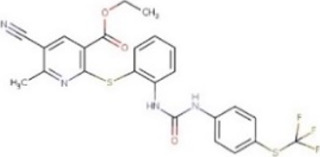	>32/>32	2.0 (1.9)	1
SPB 06643	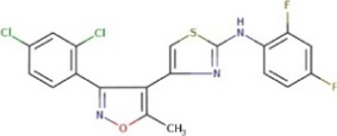	>32/>32	1.6 (1.1)	1
HTS 09153	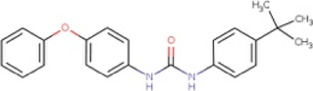	>32/>32	1.2 (2.6)	0
SPB 06551	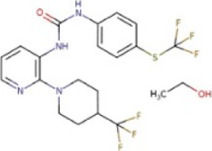	1/2	1.2 (2.4)	0
JP 00945	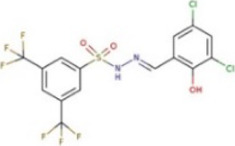	8/8	1.2 (1.60)	1

a The fold increase in final yield (OD_600_) of a *S. aureus clpX* mutant obtained in the follow-up assay, and in the original screen (value in parenthesis).

b The sensitizing score was calculated based on the summarized values given in Figure 4 (see legend to this figure for details).

c Growth completely inhibited at 4–8 mg L^−1^ SEW 02456 but limited growth observed at higher compound concentrations due to precipitation of compound.

### Minimum Inhibitory Concentration

Minimal inhibitory concentration (MIC) was determined following the Clinical and Laboratory Standards Institute 2017 guidelines in the 96-well format. Overnight cultures of *S. aureus* were diluted in physiological saline (0.9% NaCl) to reach turbidity of 0.5 McFarland (Sensititre^®^ nephelometer and the Sensititre^®^ McFarland Standard). The bacterial suspensions were adjusted to 5 × 10^5^ CFU ml^−1^ in cation-adjusted Mueller–Hinton broth in wells containing standard two-fold dilutions of the test compounds in a final volume of 100 μl. The plates were incubated for 24 h without shaking at 37°C. All experiments were performed in biological triplicates. MIC was defined as the concentration of the compounds at which visible growth was completely inhibited.

### Checkerboard Analyses and FIC Index Determination

FICs were determined by setting up checkerboard broth microdilution assays using TSB as the growth medium. Each compound and imipenem were serially diluted at eight different concentrations to create an 8 × 8 matrix. Stock solutions of BTB 00921 (5–2,500 mg L^−1^), HTS 01632 (4–2000 mg L^−1^), and BTB 04965 (5–2,500 mg L^−1^) were prepared in DMSO. While stock solutions of imipenem (50–3,200 mg L^−1^) were prepared in dH_2_O and aliquots (1.5 μl) were added to the 96-well plate. Overnight cultures of *S. aureus* were diluted in 0.9% NaCl to reach turbidity of 0.5 McFarland (Sensititre® nephelometer and the Sensititre® McFarland Standard) and 150 μl aliquots were dispensed into all wells. Plates were incubated at 37°C for 20–24 h. The FIC for imipenem in the presence of compounds (BTB 04965, BTB 00921, or HTS 01632) was calculated in wells showing <20% growth by dividing the concentration of imipenem in the presence of compound with the imipenem MIC in the absence of compound. The FIC index for the compound in combination with imipenem is the sum of the two FICs (White et al., 1996). FIC index ≤0.5 was used to show synergism. The experiment was performed in two biological replicates.

### Data Analyses

Statistical analyses were performed using R (R Core Team, 2021), and cheminformatic analyses were performed using the RDkit toolkit (https://rdkit.org) in Python 3. Pan-assay interference compounds (PAINS) were identified among the active compounds from the primary screen and the counter screen, respectively, as those compounds with a substructure matching a list of PAINS structures (https://github.com/rdkit/rdkit/blob/master/Data/Pains/wehi_pains.csv *;* Saubern et al., 2011).

## Results

### Screening Concept

**FIGURE 1:**
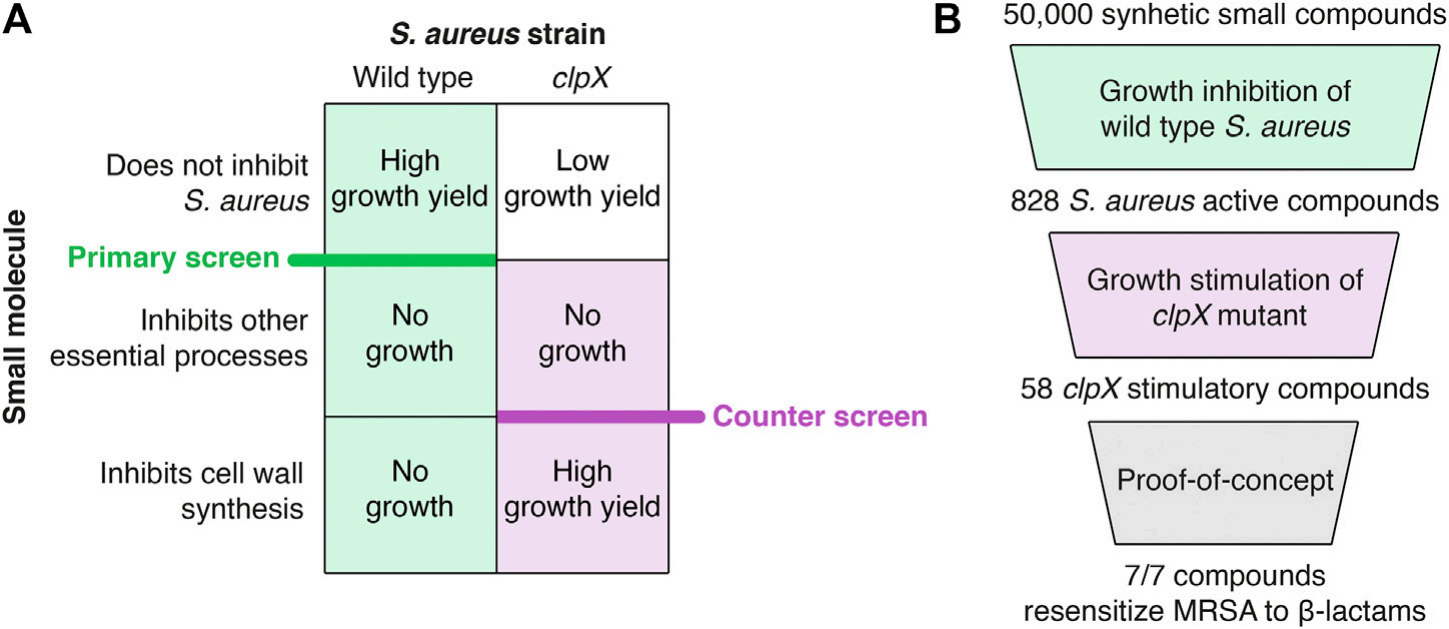
Summary of the screening procedure. **(A)** Principles of the pathway-specific screen. The screen is predicted to identify inhibitors of specific steps in cell wall synthesis because such compounds inhibit the growth of the wild type strain (primary screen) while improving growth of the *clpX* strain (counter screen)–see text for details. **(B)** Screening workflow. A collection of 50,000 synthetic small molecules from the Maybridge screening collection was first screened for growth inhibition against the *S. aureus* wild type resulting in 828 active compounds. Next, a *S. aureus clpX* mutant was used in a growth-based counter-screen to identify 58 compounds capable of increasing the growth yield of the *clpX* mutant at 30°C (cut-off 1.5 fold increase in final yield as measured by optical density). Ten compounds were purchased for follow-up studies, and of these ten compounds, seven hit-compounds retained the ability to increase the final yield of *S. aureus clpX* cultures grown at 30°C in a microtiter plate growth assay and sensitized the highly resistant COL MRSA to at least one *β*-lactam antibiotic in a disc diffusion assay (summarized in Table 1).

The screening concept is based on the findings that the cold-sensitive growth of *S. aureus clpX* mutants is rescued genetically by inactivation of LtaS, or chemically by compounds targeting TarO, catalyzing the first step in WTA synthesis, and by *β*-lactams binding to the trans peptidase domain of essential PBPs (Bæk et al., 2016; Jensen et al., 2019). TarO and LtaS are conditionally essential, and inactivation imposes a severe fitness cost at 37°C (Gründling and Schneewind, 2007; Vergara-Irigaray et al., 2008; Coe et al., 2019). We therefore reasoned that compounds targeting these crucial steps in cell wall synthesis could be identified by screening for molecules that impede growth of the wild type at 37°C, while increasing the final growth yield of the *clpX* mutant at 30°C. Hence, the screening was set up as two successive whole cell screens: a primary screen to identify compounds that inhibit growth of *S. aureus* wild type at 37°C, and a counter screen to identify compounds that increased the final OD of an *S. aureus clpX* mutant at 30°C (see overview of screen in Figure 1). Screened out compounds targeting TarO or LTA biosynthesis are predicted to sensitize MRSA strains to *β*-lactams (Campbell et al., 2011; Farha et al., 2013, Roemer et al., 2013, Lee et al., 2016).

### Primary Screen Identifies 828 Compounds Inhibiting Growth of *S. aureus*

**FIGURE 2:**
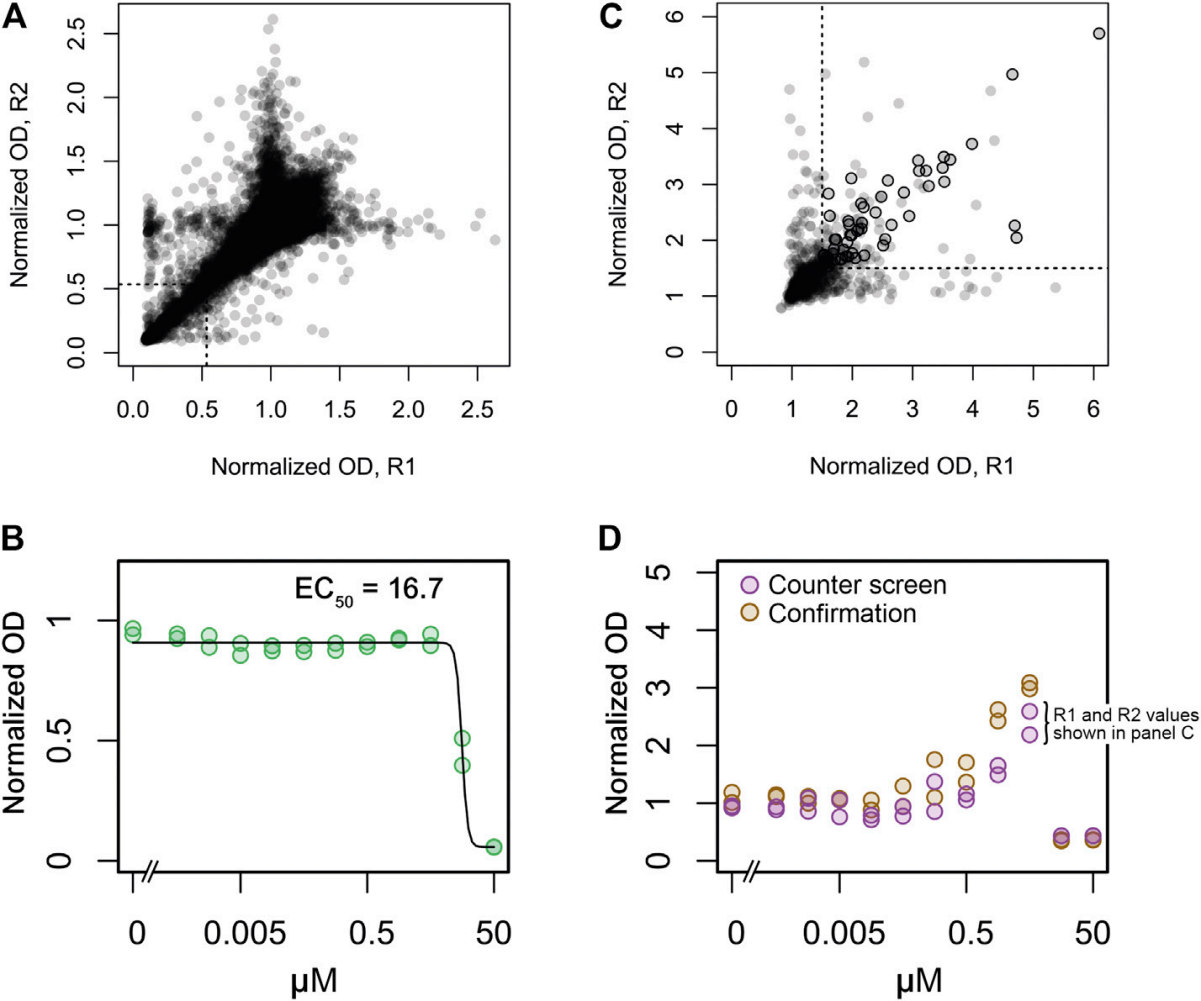
Replicate plots and hit selection for screens of growth inhibition in wild type *S. aureus* and growth stimulation in the *S. aureus clpX* mutant. **(A)** A collection of 50,000 synthetic small molecules was screened at 10 μM for growth inhibition of the wild type strain in duplicate. Normalized OD values for replicates 1 and 2 is depicted on the x- and *y*-axes, respectively. A statistical cutoff of three standard deviations below the mean was established for both replicates, indicated by the dotted lines in the lower left corner. Data points to the left and below these lines represent the 993 active compounds. **(B)** 828 of the 993 active compounds were confirmed at concentrations ranging from 0.5 nM to 50 μM in duplicate. Dose-dependent inhibition by one confirmed active compound is shown as an example. Normalized OD values for the two replicates are indicated by green circles. A calculated dose-response curve (black line) and the calculated EC50 value is also shown. **(C)** The 828 confirmed active compounds were assessed for growth stimulation of the *clpX* mutant at 30°C at concentrations ranging from 0.5 nM to 50 μM in duplicate. For each compound the highest obtained normalized OD values of replicates 1 and 2 is depicted on the *x* and *y*-axes, respectively, (these values are also indicated in panel D for one example compound). A normalized OD value of 1.5 was used as cutoff, indicated by dotted lines. Data points to the right and above these lines represent *clpX* stimulatory compounds, and black circles indicate the 58 compounds that were subsequently confirmed by manual inspection of dose-response plots and re-tests. **(D)** Dose-dependent growth stimulation by one *clpX* stimulatory compound (same compound as in panel B) is shown as an example.

The screening workflow started with a primary screen of ∼50,000 small synthetic compounds from the Maybridge screening collection for growth inhibition of wild type methicillin sensitive *S. aureus* (strain SA564) at a concentration of 10 μM. Growth at 37°C was measured by change in absorbance (600 nm) after 8 h of incubation with no shaking in 384-well plates. These conditions led to an optimal screening window at late exponential growth phase (Supplementary Figure 1A). Throughout the screen, high (1% DMSO) and low (2.5 mg L^−1^ erythromycin) controls were included. The screening data were normalized to remove plate-to-plate and well-positional variation (Mangat et al., 2014). Hits were selected as those molecules causing the normalized OD values to be lower than three standard deviations below the mean of the full data set resulting in a hit rate of 2.0% and a total of 993 *S. aureus* active compounds (Figure 2A). Of these, 828 were confirmed as active when tested in 11 different concentrations ranging from 0.5 nM to 50 μM (Figure 2B).

### Counter Screen Identifies 58 Compounds That Rescue Growth of *S. aureus clpX* Mutant

This sub-library of 828 compounds with confirmed growth-inhibitory activity against *S. aureus* was then used as a starting point in a counter-screen for growth stimulation of the *S. aureus clpX* mutant at 30°C at 11 different concentrations of each compound ranging from 0.5 nM to 50 μM. Growth was measured by a change in absorbance (600 nm) after 24 h of incubation with shaking at 600 rpm in 96-well plates. These conditions led to an optimal screening window when we tested screening conditions with oxacillin at a concentration (0.05 mg L^−1^) previously shown to stimulate growth of the *clpX* mutant (Jensen et al., 2019; Supplementary Figure 1B). The screening data were normalized to remove plate-to-plate variation as described in *Methods.*

In the counter-screen, a compound was classified as active if it raised the final growth yield of the *clpX* mutant compared to the DMSO control by 1.5 fold or more. This cutoff-value immediately resulted in a set of 678 inactive compounds that were discarded from further analyses (Figure 2C). The dose-response plots of the remaining compounds were then inspected manually, and the compounds were classified as either inactive, active, or inconclusive (42 compounds, Figure 2D). Fifty of the compounds were also re-tested using the same assay. In total, 58 (7%) of the 828 compounds (or 0.12% of all screened compounds) that inhibited *S. aureus* growth also stimulated *clpX* growth with maximal growth yield increases ranging from 1.5 to 3.7-fold.

### The Counter Screen is Efficient at Eliminating Compound Classes That Tend to Have Non-Specific Activities

An important advantage of employing a counter screen selecting for improved growth is that nonspecific growth inhibitors are likely to be eliminated from the pool of hit compounds. A class of compounds that often show up as hits in screening campaigns, are *pan-assay interference compounds* (PAINS) which are chemical compounds that tend to react nonspecifically with numerous biological targets rather than specifically affecting one desired target (Baell and Holloway, 2010). Applying an *in silico* PAINS filter to the 58 hits shows that only two of the final hit compounds (3%) contain a PAINS substructure, whereas this is the case for 9% of the *S. aureus* growth-inhibitory compounds that do not stimulate *clpX* growth. This result indicates that the *clpX* counter-screen is efficient at eliminating compound classes that tend to have non-specific activities.

### Hit Compounds Reverse *β*-lactam Resistance in MRSA

**FIGURE 3:**
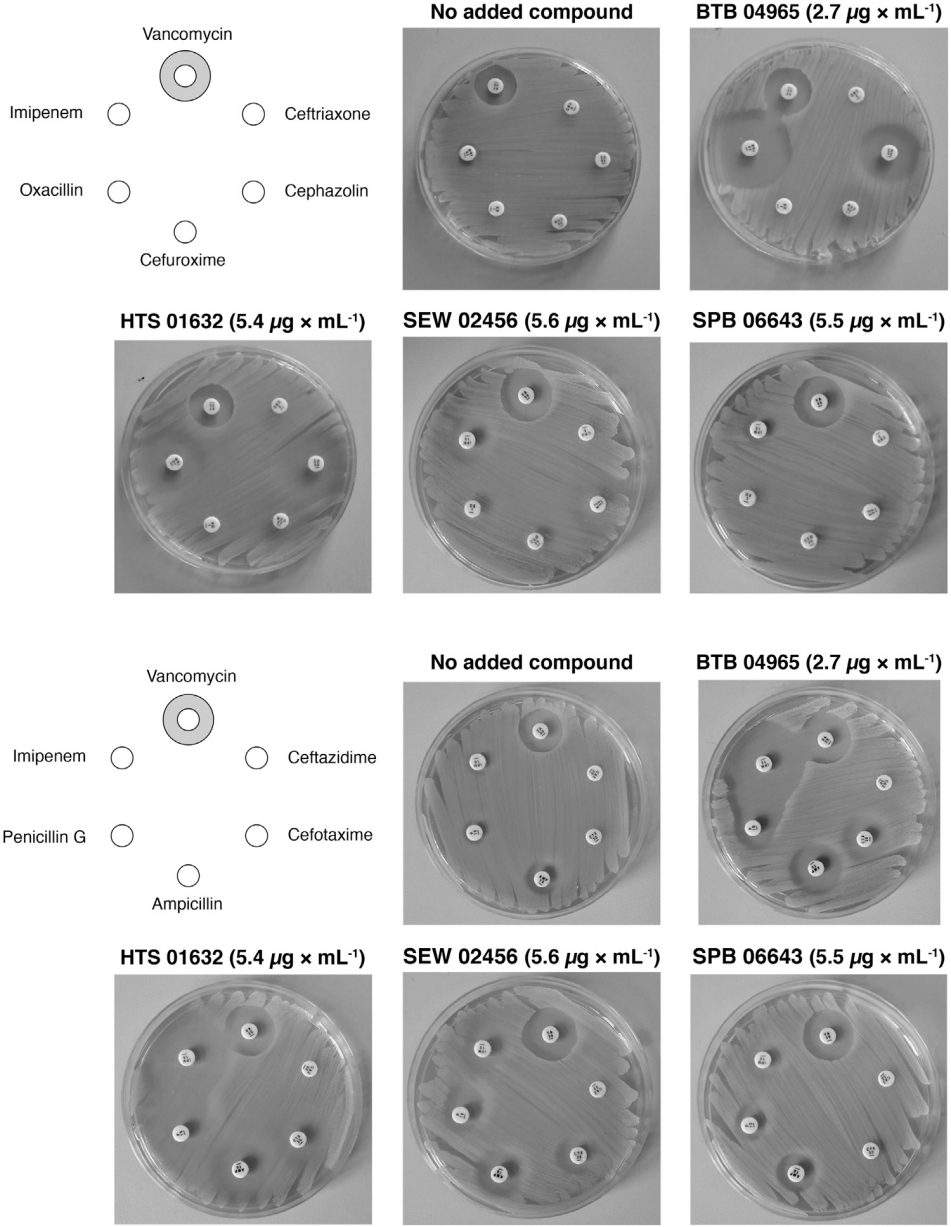
Reversal of *β*-lactam resistance in MRSA strain COL by addition of hit-compounds. The sensitivity of the COL MRSA strain towards different *β*-lactams and vancomycin (negative control) in the absence or presence of hit-compounds was examined by a disc diffusion assay. Results of the disc diffusion assay performed with four compounds with decreasing ability to increase the growth yield of *S. aureus clpX* mutants are shown. The hit-compounds were added to the agar at the indicated sub-lethal concentrations, and antibiotic susceptibility discs were placed on a lawn of the MRSA strain COL as indicated on the left diagram.

**FIGURE 4:**
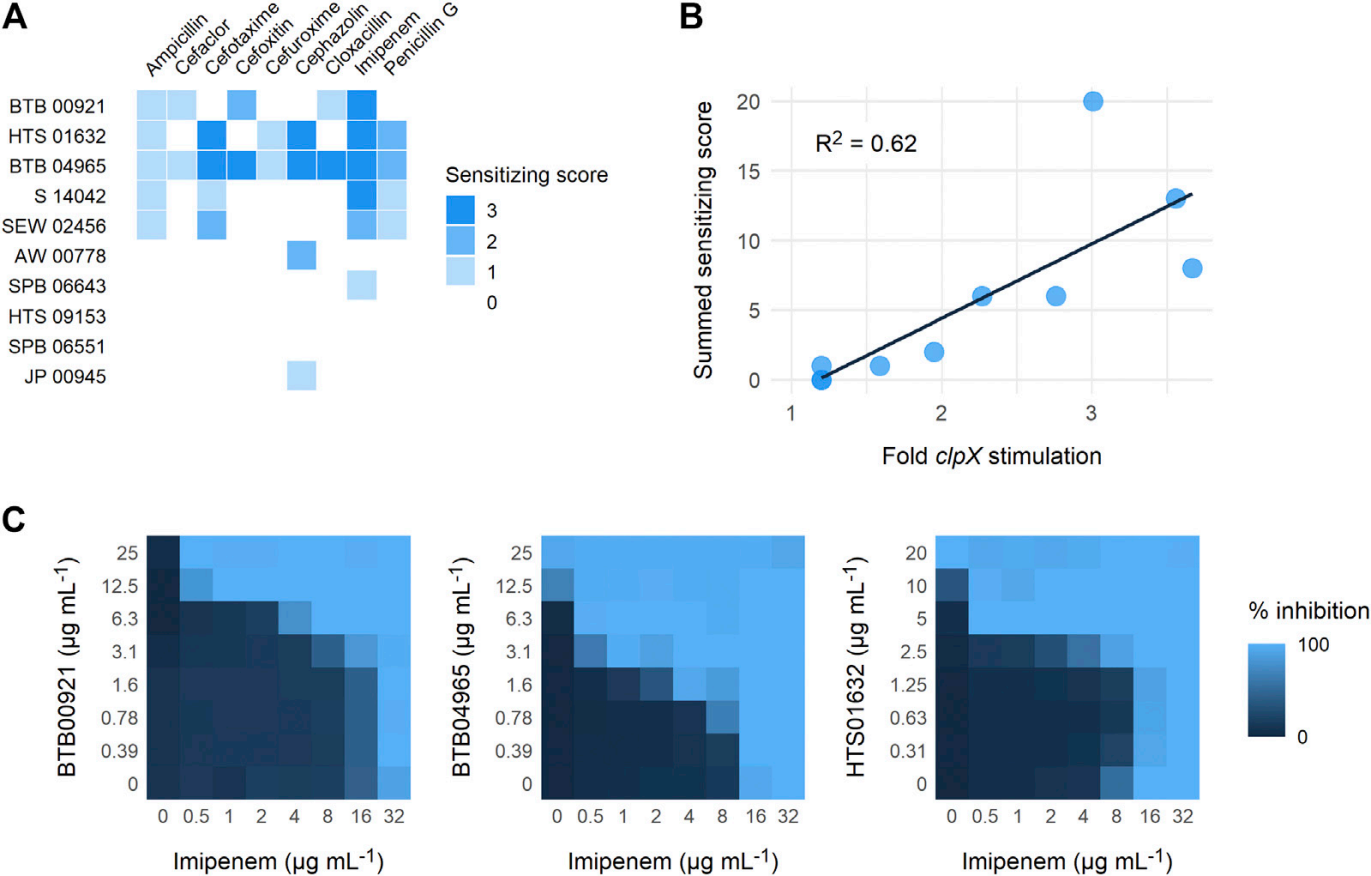
Reversal of *β*-lactam resistance in MRSA strain COL. **(A)** The diameter of the clearing zones in the disc diffusion assay was measured and the ratio of the diameters of the inhibition zones in the presence and absence of compound were used to calculate a sensitizing score for each compound/*β*-lactam combination as described in the *Methods* section. The compounds are listed according to their ability to stimulate growth of the *clpX* mutant from bottom to top. **(B)** The scores across all antibiotics are added to give a total synergy score for each compound. The score is plotted against the fold change in growth of *clpX* in the presence of compound, together with a linear regression line (*R* ^2^ = 0.62) **(C)** Synergy between imipenem and the three compounds with highest sensitizing scores was evaluated by performing microdilution checkerboard analyses against the highly resistant MRSA strain, COL. The extent of inhibition is shown as a heat plot.

To establish a proof-of-concept, we purchased a subset of ten screening compounds and tested them for their ability to sensitize MRSA to *β*-lactams. The ten compounds were chosen based on their varying ability to stimulate growth of the *S. aureus clpX* strain, with the ten compounds ranking from showing no stimulation (below the 1.5 cut-off) to maximal stimulation (3.6 fold increase in final OD) in the screening set-up. We first examined if the hit compounds retained the ability to increase the growth yield of *clpX* cells by measuring the final OD (600 nm) reached by the SA564 *clpX* mutant after 24 h of incubation in the absence or presence of added compounds. Seven compounds met the 1.5 fold stimulation cut-off used in the secondary screen, and, in general, there was good correlation between the fold stimulation observed in this assay and the fold-stimulation determined in the original screening assay (Table 1). However, one compound (SPB 06643), which did not meet the cut-off of 1.5 fold stimulation in the original screen, showed a minor (1.6 fold) stimulation in this assay, while two compounds did not meet the 1.5 fold stimulation cut-off. We then examined the ability of the ten compounds to inhibit growth of wild type cells by determining the MICs against two different *S. aureus* wild type strains, the methicillin sensitive SA564 strain, which was used in the primary screen, and the JE2 MRSA strain belonging to the fast spreading and highly virulent community-acquired USA300 clone (Fey et al., 2013). As can be seen in Table 1, the MIC values for the compounds varied from 1 to 2 mg L^−1^ to exceeding 32 mg L^−1^ with similar MIC values measured against the JE2 MRSA strain and the methicillin sensitive SA564 strain. The high MIC values are in line with the potential inhibition of non-essential targets such as TarO. Next, we assessed if the compounds had the ability to sensitize the highly resistant MRSA strain COL to *β*-lactam antibiotics by doing a disc diffusion assay. In the absence of added compounds, COL displayed high resistance to all tested *β*-lactams antibiotics as evidenced by the absence of clearing zones surrounding the antibiotic discs (Figure 3). Remarkably, enlarged inhibition zones for one or more *β*-lactams was observed in the presence of sub-lethal concentrations of the seven compounds that met the 1.5 fold cut-off in the follow-up stimulation assay, demonstrating that these compounds are capable of sensitizing the MRSA strain to *β*-lactams (see specific examples in Figure 3, and a summary of the results in Table 1). The five most potent compounds sensitized the COL strain to four or more different types of *β*-lactams (Figure 4A). Notably, when we used the fold increase in the diameter of the inhibition zones in the presence and absence of compound to score the sensitizing effect for each compound/*β*-lactam combination (see *Methods* for details and illustrated in Figure 4A) the summed sensitizing scores for each compound correlated linearly to the fold-stimulation of the SA564 *clpX* strain (Figure 4B). Therefore, the degree of growth stimulation of the *clpX* mutant seems to be a good predictor of a compound’s ability to reverse *β*-lactam resistance. In the disc diffusion assay, the strongest sensitizing effect was observed for imipenem in combination with BTB 00921, HTS 01632, and BTB 04965 (Figure 4A and Table 1). To more directly quantify the sensitizing effect, we finally performed checkerboard analyses for imipenem in combination with each of these three compounds (Figure 4C). We found that imipenem MIC was reduced up to 64-fold in the presence of sub-inhibitory concentrations of BTB 00921, HTS 01632, or BTB 04965 (Figure 4C). Two compounds display synergy if the fractional inhibitory concentration (FIC) index, as calculated by the sum of the FIC of each compound, is ≤0.5 (White et al., 1996). According to this definition, imipenem has synergy with BTB 04965 (average FIC index = 0.4 with FIC index ranging from 0.19 to 1.0 in single wells) and with HTS 01632 (average FIC index = 0.5 with FIC index ranging from 0.28 to 1.0 in single wells). The FIC index for BTB 00921 in combination with imipenem could not be calculated as BTB 00921 does not reach the MIC. In conclusion, our results demonstrate the efficiency of the screening setup in identifying hit-compounds that sensitize MRSA to *β*-lactams antibiotics.

## Discussion

The cell wall continues to be an excellent target for antibacterial drug discovery because of its essentiality in bacteria and its absence in mammalian cells. In this report, we describe the development and implementation of a high-throughput screening approach where a *S. aureus* mutant lacking the ClpX chaperone was used in a counter-screen to identify presumed cell wall synthesis inhibitors that at sub-inhibitory concentrations sensitize MRSA to *β*-lactams antibiotics. These hit-compounds could potentially be used in combination therapy with *β*-lactams for treatment of MRSA-infections. Additionally, some of the hit-compounds show inhibitory activity against *S. aureus* at therapeutic relevant concentration (1–2 µM) and, hence, hold potential for being developed into lead compounds for mono-therapy of staphylococcal infections. However, follow up studies are needed to pin-point the precise target of the hit-compounds. Based on the findings that 1) growth of *clpX* cells is very specifically rescued by compounds targeting TarO, PBP1 or PBP3 (Jensen et al., 2019), and that 2) spontaneous suppressor mutations only mapped in *ltaS*, we predicted that hit-compounds would directly or indirectly target a pathway that functionally connect TarO, PBP1/PBP3, and LtaS. So far, the molecular mechanism underlying the dramatic synergy between *β*-lactams and TarO inhibitors against MRSA remain unexplained. Interestingly, we here observed very good correlation between the ability of the hit-compounds to increase the growth yield of the *clpX* mutant, and the ability of the compounds to sensitize MRSA to *β*-lactam antibiotics. The sensitizing effect varied widely between different types of *β*-lactams as was previously shown for TarO inhibitors (Campbell et al., 2011; Farha et al., 2013). For all compounds, the strongest sensitizing effect was observed with imipenem that is specific for *S. aureus* PBP1 whose function is confined to synthesis of the septal wall (Reichmann et al., 2019). Strikingly, imipenem is also superior to other *β*-lactams in improving growth of *clpX* cells (Jensen et al., 2019). Taken together, these correlations point to a functional connection between the early steps of WTA biosynthesis and the transpeptidase domain of PBPs that is critical for both the synergy between TarO inhibitors and *β*-lactams, and for alleviating the cold-sensitive growth defect of *clpX* cells. Inactivation of *clpX* results in accumulation of the Sle1 cell wall hydrolase involved in separation of *S. aureus* daughter cells (Thalsø-Madsen et al., 2019). The severe growth defect of *clpX* cells was explained by showing that at 30°C, a combination of aberrant septum synthesis and high Sle1 levels caused premature splitting of daughter cells resulting in cell lysis (Jensen et al., 2019). Remarkably, *β*-lactams prevented Sle1 dependent lysis of *clpX* cells (Jensen et al., 2019). As also WTA and LTA have a crucial role in promoting septal localization of autolysins, the ability to antagonize Sle1 mediated lysis could be a central feature in providing *clpX* stimulation (Schlag et al., 2010; Zoll et al., 2012). Therefore, our *clpX* based counter screen may select broadly for compounds that impede autolytic splitting of daughter cells. The mechanisms coordinating cell wall hydrolase activity with peptidoglycan synthesis are crucial for bacterial viability, however, relatively little is known about the check points that safeguard bacteria from the detrimental activity of cell wall hydrolases during the cell cycle. We hope that a further characterization of the hit-compounds identified in this study will bring novel insight into these important mechanisms.

## Data Availability

The original contributions presented in the study are included in the article/Supplementary Material, further inquiries can be directed to the corresponding author.
