# Supplementary figures and images for: A Staphylococcus aureus clpX Mutant Used as a Unique Screening Tool to Identify Cell Wall Synthesis Inhibitors that Reverse β-Lactam Resistance in MRSA
Source: Front Mol Biosci. 2021 Jun 4;8:691569. doi: 10.3389/fmolb.2021.691569 (PMC8212132; doi:10.3389/fmolb.2021.691569)

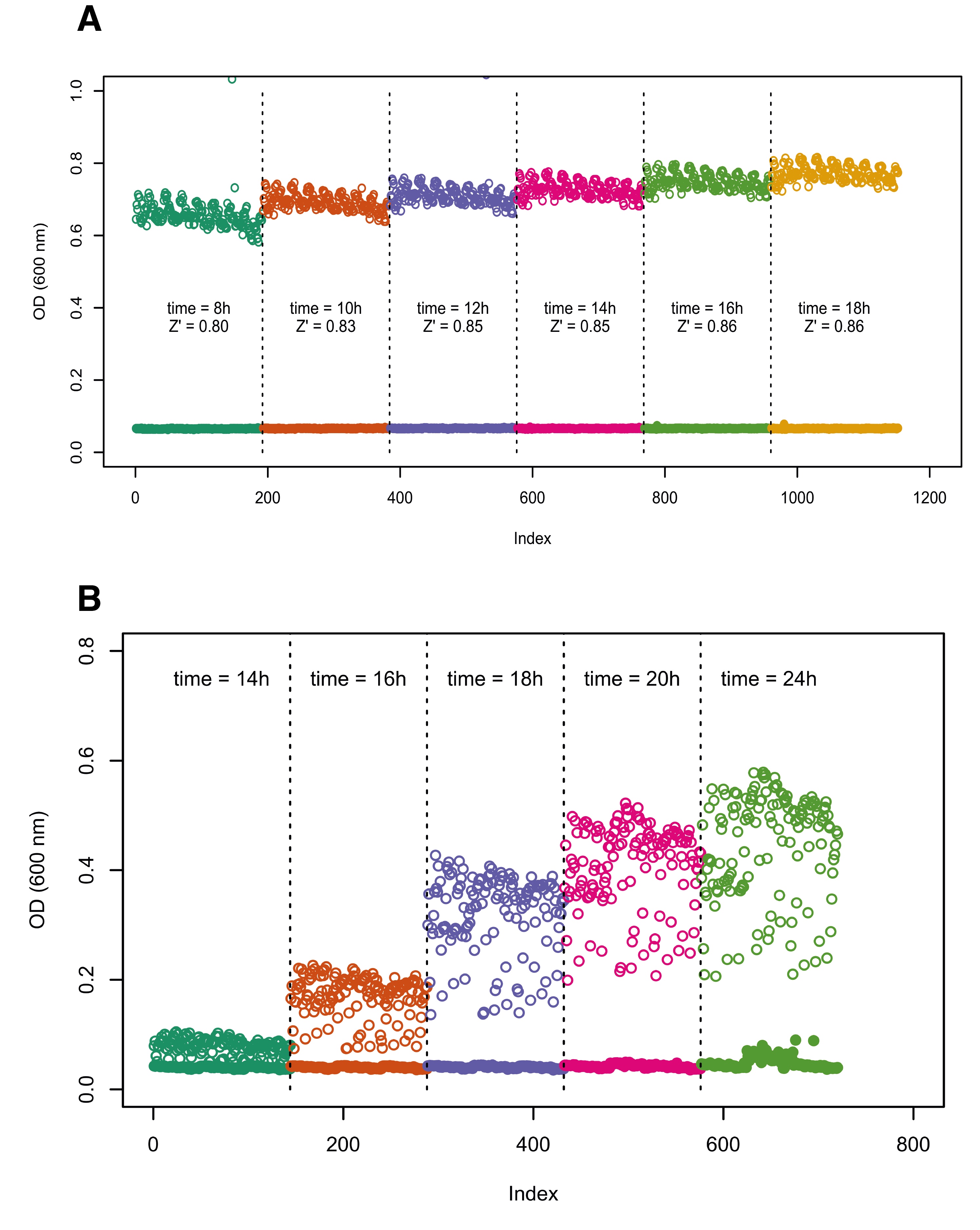

Supplement: Supplementary file 1 [file Image1.JPEG]
